# Prediction of In Vivo Knee Mechanics During Daily Activities Based on a Musculoskeletal Model Incorporated with a Subject-Specific Knee Joint

**DOI:** 10.3390/bioengineering12020153

**Published:** 2025-02-05

**Authors:** Li Zhang, Hui Li, Xianjie Wan, Peng Xu, Aibin Zhu, Pingping Wei

**Affiliations:** 1 Honghui Hospital, Xi’an Jiaotong University, Xi’an 710054, China; xjtuzhangli@xjtu.edu.cn (L.Z.); drlihuiortho@gmail.com (H.L.); wxj951216@163.com (X.W.); 2 Shaanxi Key Laboratory of Intelligent Robots, Institute of Robotics and Intelligent Systems, Xi’an Jiaotong University, Xi’an 710049, China; abzhu@mail.xjtu.edu.cn; 3 State Key Laboratory for Manufacturing Systems Engineering, Xi’an Jiaotong University, Xi’an 710054, China; erin.wei@mail.xjtu.edu.cn

**Keywords:** subject-specific knee model, gait analysis, musculoskeletal model, tibiofemoral contact force

## Abstract

The objective of this study was to develop a musculoskeletal model incorporated with a subject-specific knee joint to predict the tibiofemoral contact force (TFCF) during daily motions. For this purpose, 18 healthy participants were recruited to perform the motion data acquisition using synchronized motion capture and force platform systems, and motion simulation based on an improved musculoskeletal model for five daily activities, including normal walking, stair ascent, stair descent, sit-to-stand, and stand-to-sit. The proposed musculoskeletal model included subject-specific models of bones, cartilages, and meniscus, detailed knee ligaments and muscles, deformable elastic contacts, and multiple degrees of freedom (DOFs) of the knee joint. The prediction accuracy was demonstrated by the good agreements of TFCF curves between the model predictions and in vivo measurements for the five activities (RMSE: 0.216~0.311 BW, R^2^: 0.928~0.992, and C_E_: 0.048~0.141). Based on the validated model, the TFCF on total, medial, and lateral compartments (TFCF_Total_, TFCF_Medial_, and TFCF_Lateral_) during the five daily activities were predicted. For TFCF_Total_, the peak force for stair descent or sit-to-stand was the largest, followed by stair ascent or stand-to-sit, and finally normal walking. For TFCF_Medial_, stair descent had the largest peak, followed by stair ascent. There were no significant differences between the peak TFCF_Medial_ values of normal walking, sit-to-stand, and stand-to-sit. For TFCF_Lateral_, the peak of sit-to-stand was the largest, followed by stand-to-sit or stair descent, and finally normal walking or stair ascent. This study is valuable for further understanding the biomechanics of a healthy knee joint and providing theoretical guidance for the treatment of knee osteoarthritis (KOA).

## 1. Introduction

As a prevalent, chronic, degenerative, and multifactorial disease, knee osteoarthritis (KOA) has become a significant public health problem in the world [1]. Epidemiological studies show that the global prevalence of KOA was 16.0% (19.2% in Asia and 13.4% in Europe) [2]. For people over the age of 60, the global prevalence increased to 50.1% in women and 20.2% in men [3]. Abnormal load distribution has been designated as the main mechanical factor leading to KOA [4]. Therefore, quantification of internal knee contact force during daily activities is important in comprehending KOA initiation and progression, and improving KOA treatments, such as total knee arthroplasty (TKA), knee orthoses, gait training, etc.

As an effective method for the quantitative analysis of internal knee contact force, specifically tibiofemoral contact force (TFCF), gait analysis based on motion data acquisition using multiple cameras and force platforms and motion simulation using the musculoskeletal model have been widely used in recent years [5]. Increasing the personalization of the musculoskeletal model has been considered as an important method for enhancing prediction accuracy [6]. Lerner et al. [7] incorporated the moveable contacts of knee medial and lateral compartments and subject-specific lower-limb alignment into their model. Zeighami et al. [8] presented a model with subject-specific knee contact locations. Nonetheless, subject-specific bone, one-degree-of-freedom (DOF) knee joint, and rigid point-to-point contacts were applied. Moissenet et al. [9] and Barzan et al. [10] introduced length-variable ligaments and magnetic resonance imaging (MRI)-based sphere-on-sphere knee contact into their musculoskeletal model. But one-DOF knee joint and idealized rigid contacts still limited the personalization of the model. Smith et al. [11] proposed a model that included deformable contact, ligamentous structures, and a multi-DOF knee joint. However, their musculoskeletal model utilized linear scaling to scale the bone models. Kia et al. [12] incorporated personalized bone geometry, deformable elastic contact condition, ligaments, and 12 DOFs into their knee model. Nevertheless, the prediction accuracy depended on the setting of global control gains. According to the force-dependent kinematics (FDK) algorithm [13], a model with knee ligaments, deformable contacts, subject-specific bones, and a multiple-DOF knee joint was established by Marra et al. [14]. But the model was only able to predict the TFCF for patients after TKA. Nearly all of the above studies focused on only one activity, namely, normal walking. To our knowledge, there are few studies that simultaneously predict the TFCF during various daily activities. Konrath et al. [15] adopted the generic musculoskeletal model in AnyBody software to predict the TFCF during stair ascent, stair descent, and sit-to-stand. Wan et al. [16] utilized the generic musculoskeletal model in Visual3D software to estimate the TFCF during normal walking, stair ascent, stair descent, sit-to-stand, and stand-to-sit. Based on the musculoskeletal model of Lerner et al. [7], Valente et al. [17] predicted the TFCF during normal walking, stair ascent, and stair descent. At present, there is no research on the prediction of TFCF during various daily activities based on the musculoskeletal model incorporated with a subject-specific knee joint.

The aim of the present study was to (a) develop a musculoskeletal model incorporated with a subject-specific knee joint; (b) assess the prediction accuracy by comparing the TFCF curves between the model predictions and in vivo measurements; (c) predict the TFCF on the total, medial, and lateral compartments (TFCF_Total_, TFCF_Medial_, and TFCF_Lateral_) during daily activities. In this study, normal walking, stair ascent, stair descent, sit-to-stand, and stand-to-sit were selected because they are essential and frequent daily activities [18,19,20]. We hypothesized that our proposed musculoskeletal model incorporated with a subject-specific knee joint may predict the TFCF during the five daily activities with reasonable accuracy.

## 2. Materials and Methods

### 2.1. Participants

In this study, 18 healthy participants (sex (male/female): 9/9, height: 1.78 ± 0.08 m, weight: 73.4 ± 6.8 kg, age: 22 ± 8 yrs., and body mass index (BMI): 22.9 ± 2.3 kg/m^2^) were recruited. A sample size was calculated with G*Power software (version 3.1.9.7, Heinrich Heine University, Dusseldorf, Germany). According to the effect size reported by Van Rossom et al. [21] and the results of the pilot study, to achieve a power of 0.8 with α at 0.05, the ideal sample size was 15. All participants were recruited from our university. The inclusion criteria were as follows: (1) age ≥ 18 years and (2) ability to walk independently with no limitations. The exclusion criteria were as follows: (1) a BMI > 30 kg/m^2^, (2) any history of lower-limb injuries, and (3) any history of neuromuscular disease. Screenings were conducted through physical examinations, and all participants met the eligibility criteria. Ethical approval was obtained from the Ethics Committee of Honghui Hospital, Xi’an Jiaotong University (No: 202309006 and date of approval: 9 October 2023). Before data collection, the purpose of this study and the procedures involved were fully explained to each participant, and written informed consent was signed before their enrollment. All methods of this study were performed in accordance with the Declaration of Helsinki.

### 2.2. Data Acquisition

**Figure 1:**
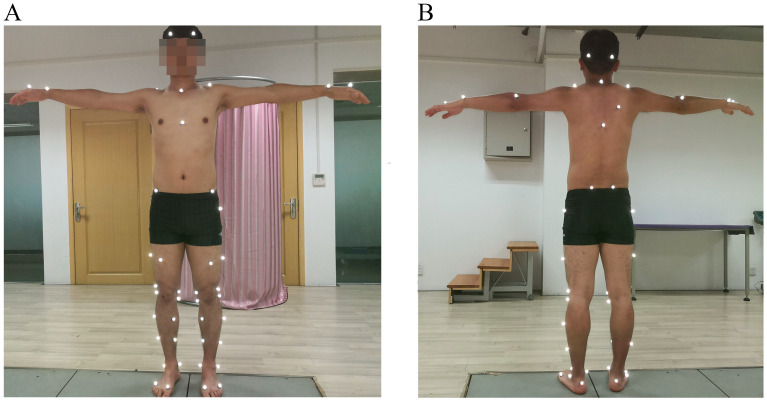
Positions of the 55 reflective markers, (**A**) frontal view and (**B**) back view.

Three-dimensional (3D) data during static and dynamic conditions were collected using a 10-camera motion capture system (Vicon, Oxford, UK). The sampling frequency of the system was 100 Hz. As shown in Figure 1, 55 reflective markers (15 mm) were attached to the full body of each participant based on our previously published marker setting [22]. Four markers were attached bilaterally onto the anterior superior iliac spine and posterior superior iliac spine to form the pelvic segment. Thirty-two markers were attached bilaterally to the greater trochanter, lateral thigh, anterior thigh, medial and lateral femoral epicondyles, medial and lateral tibial condyles, medial and lateral superior shank, medial and lateral inferior shank, medial and lateral malleolus, heel, toe, and 5th metatarsal to form the right and left lower limb segments. Four markers were attached bilaterally onto the front head and back head to form the head segment. Seven markers were attached onto the 7th cervical vertebrae, 10th thoracic vertebrae, jugular notch, xiphoid process, middle of scapula, and bilateral shoulders to form the trunk segment. Eight markers were attached bilaterally onto the lateral humerus epicondyle, wrist bar thumb side, wrist bar pinkie side, and 2nd metatarsal of hand to form the right and left upper limb segments. Three-dimensional ground reaction forces (GRFs) were record using a 3-force platform system (AMTI, Watertown, MA, USA). The sampling frequency of the system was 1000 Hz. The motion capture system and the 3-force platform system were connected to an MX data conversion console through an MX connection line to realize synchronous data acquisition.

Before data acquisition, each participant familiarized themselves with the tasks and warmed up, including normal walking, stair ascent, stair descent, sit-to-stand, and stand-to-sit, with a self-selected comfortable speed. The total time for each participant to familiarized themselves with the tasks and warmed up was about 30 min. After they warmed up, static and dynamic data collections were conducted for each participant. During the static data collection, the participant was asked to stand upright with their feet placed shoulder-width apart. The static data were important for the scaling of the musculoskeletal model, except for the subject-specific knee joint model. During the dynamic data collection, the participant randomly performed the five daily activities at a self-selected speed. In this study, a 15 m walkway, a 3-step staircase (0.29 m tread and 0.17 m riser), and a 0.44 m seat were utilized for normal walking, stair ascent/descent, and sit-to-stand/stand-to-sit, respectively. During each of the activities, at least 20 repetitions were performed. To remove the effects of fatigue, a 5 min rest period was provided between two tasks.

Task completion was defined as follows: the period from heel strike to the next heel strike of the same foot for normal walking; from foot–stair contact to the next contact of the same foot for stair ascent and stair descent; from the moment the buttocks leave the seat to the standing posture for sit-to-stand; and from the standing posture to buttocks contact with the chair for stand-to-sit. For normal walking, stair ascent, and stair descent, the events were detected based on the 3D GRF data by using the event detection algorithm in Vicon Nexus software (version 2.6.1, Vicon, Oxford, UK). For sit-to-stand and stand-to-sit, the events were detected based on the methods outlined in the study of Etnyre et al. [23].

**Figure 2:**
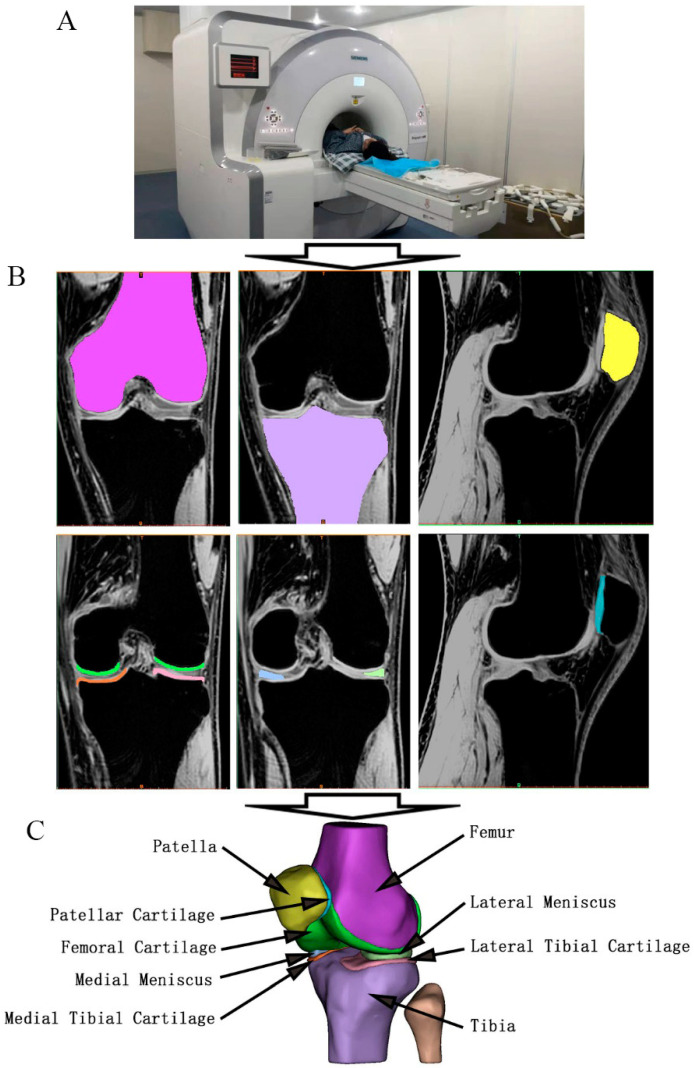
The 3D geometric modeling of bones, cartilages, and meniscus, (**A**) the MRI scanner, (**B**) the raw tomographic images, and (**C**) the 3D geometric models.

As shown in Figure 2A, A 3.0 T MRI scanners (SIEMENS, Munich, Germany) and an 8-channel knee coil were applied to acquire the geometric data of bones, cartilages, and meniscus. The participant was positioned supine, with the patella facing upward for the MRI scan. The MRI scans were performed in the sagittal plane, with the following parameters: a Dual Echo Steady State (DESS) sequence, a slice thickness of 1.0 mm, an interslice spacing of 0 mm, a matrix size of 512 pixels × 512 pixels, a field of view (FOV) of 16 cm, a repetition time (TR) of 15 ms, and an echo time (TE) of 5 ms. The raw MRI data were stored in DICOM format.

Then, the raw MRI data were imported into the Mimics software (version 21.0, Materialis NV, Leuven, Belgium) to reconstruct the 3D geometric models (see Figure 2B). Firstly, based on the differences in gray values between the different components in MRI data, the masks of each bone, cartilage, and meniscus were manually extracted layer by layer by using the “Masks” and “Dynamic Region Growing” features in the software to realize image segmentation between the different components of the knee joint. Secondly, 3D reconstruction was performed to obtain the 3D models of the various components of the knee joint by using the “3D Calculate” feature in the software. Thirdly, the smooth optimization of each component of the knee joint model was carried out by using the “Smooth” and “Warp” features in the software on the premise of ensuring the correct 3D geometric structure and not losing geometric details. Finally, the 3D model of each component of the knee joint was stored in STL format.

Although the 3D models in STL format generated by Mimics software were smooth-optimized, there were still some nail surfaces or overlapping surfaces on the local surface of the models. Therefore, the Geomagic Studio software (version 2014, Geomagic, NC, USA) was employed to further perform smooth optimization on the premise of ensuring the correct 3D geometric structure and not losing geometric details, including removing noise, simplifying the number of surfaces, repairing small grids and cusps, surface reconstruction, and surface fitting. The processed models are shown in Figure 2C. The 3D models after the above processing were imported into the HyperMesh software (version 2021, Altair, Troy, MI, USA) for remeshing with the element type of C3D4. The mesh density was set to 4 mm for bone, 1 mm for cartilage, and 0.8 mm for meniscus, based on the verified and published setting [24].

### 2.3. A Musculoskeletal Model Incorporated with the Subject-Specific Knee Joint

**Figure 3:**
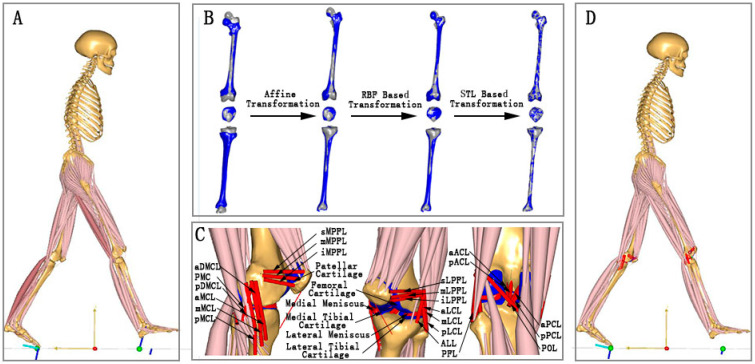
The musculoskeletal modeling incorporated with a subject-specific knee joint, (**A**) the generic musculoskeletal model, (**B**) the subject-specific morphing of femur, tibia, and patella, (**C**) the ligaments and contacts modeling, and (**D**) the musculoskeletal model incorporated with a subject-specific knee joint.

The musculoskeletal model proposed in this study was based on the generic musculoskeletal model in AnyBody software (version 7.2, Aalborg, Denmark). As shown in Figure 3A, the knee joint of the generic model does not include the knee ligaments, cartilages, and meniscus. Details about the generic model can be found in reference [25].

For the femur, tibia, and patella, an advanced morphing method was utilized to enhance the personalization of bone geometry (Figure 3B) [26]. Firstly, the bones were linearly scaled by using the affine transformation. Secondly, local nonlinear deformation was performed to morph the local bone in detail through the radial basis function (RBF)-based interpolation/extrapolation transformation. Thirdly, an STL-surface-based transformation was used to improve the nonlinear morphing of the bone surface. In addition, the remaining bones were linearly scaled based on the length–mass–fat scaling law. After bone scaling, the models of cartilages and meniscus were aligned with the related bones (Figure 3C).

Ten ligaments (22 bundles) surrounding the knee joint were modeled in this study (Figure 3C and Table 1). The attachment sites of each ligament bundle were quoted from previous studies [27,28] and linearly scaled based on the length–mass–fat scaling law. The ligament bundles were represented by the following force–strain ($f^{L}$-ε) relationship [29]:

(1) $$\begin{array}{c} f^{L}=\left\{\begin{array}{c} \begin{array}{cc} 0 & \varepsilon <0 \end{array}\\ \begin{array}{cc} 1/4k\varepsilon^{2}/\varepsilon_{l} & 0\leq \varepsilon \leq 2\varepsilon_{l} \end{array}\\ \begin{array}{cc} k\left(\varepsilon -\varepsilon_{l}\right) & \varepsilon >2\varepsilon_{l} \end{array} \end{array}\right. \end{array}$$

where *k*, ε, and $\varepsilon_{l}$ are the reference linear stiffness, strain, and linear strain limit, respectively. *k* and $\varepsilon_{r}$ (strain in full knee extension) were selected as shown in Table 1.

For the muscle modeling, the maximum isometric force (*F*_0_) of each muscle tendon unit (MUT) was calculated by the following:

(2) $$\begin{array}{c} F_{0}=\mu \times PCSA=\mu \times \frac{{Lf}_{0}}{{Vol}_{0}} \end{array}$$

where *PCSA*, ${Lf}_{0}$, and ${Vol}_{0}$ represent the physiological cross-sectional area, optimum fiber length, and muscle volume, respectively. The symbol μ indicates the factor of *PCSA* and was set to 27 $N/{cm}^{2}$ [31]. The length–mass–fat scaling law was applied to linearly scale the muscle strength. For muscle recruitment, a cubic polynomial optimization method was employed [32]:

(3) $$\begin{array}{cc} Minimize & G\left(f^{M}\right)=\sum_{i=1}^{n}V_{i}{\left(\frac{{f_{i}}^{M}}{N_{i}}\right)}^{3}\\ subject\;to & Cf=r\\ & {f_{i}}^{M}\geq 0\;\;i=1,\cdots ,\;n \end{array}$$

where *G* is the objective function, ${f_{i}}^{M}$, $N_{i}$, and $V_{i}$ are the force, instantaneous strength, and volume weight factor of the *i*th muscle bundle, respectively. The symbol ***C*** stands for the coefficient matrix. All the muscle forces, ligament forces, joint contact forces, and the residual forces are indicated as ***f***. And all the external forces and inertia forces are represented as ***r***.

Five deformable elastic contact pairs (medial/lateral tibial cartilage and femoral cartilage, medial/lateral meniscus and femoral cartilage, and patellar cartilage and femoral cartilage) were defined in our model (Figure 3C). For each contact pair, the contact surfaces were meshed into STereoLithography (STL) triangles. The magnitude of $F_{i}$ (contact force at the *i*th vertex) was calculated according to the linear force–penetration volume law [14]:

(4) $$F_{i}=P_{M}V_{Pi}=P_{M}A_{i}d_{i}$$

where $V_{Pi}$, $A_{i}$, and $d_{i}$ are the penetration volume, triangle area of the opposite surface, and penetration depth of the *i*th vertex, respectively. The pressure module ($P_{M}$) was computed by the following [33]:

(5) $$P_{M}={\left(\frac{\left(1+v_{1}\right)\left(1-2v_{1}\right)h_{1}}{\left(1-v_{1}\right)E_{1}}+\frac{\left(1+v_{2}\right)\left(1-2v_{2}\right)h_{2}}{\left(1-v_{2}\right)E_{2}}\right)}^{-1}$$

where $v_{1}$ and $v_{2}$, $E_{1}$ and $E_{2}$, and $h_{1}$ and $h_{2}$ indicate Poisson’s ratio, the elasticity modulus, and the average elastic layer thickness of the two contact objects, respectively (see Table 2).

In our study, a 11-DOF knee joint (6 DOFs for the tibiofemoral joint and 5 DOFs for the patellofemoral joint) was utilized. In inverse dynamics, the flexion/extension of tibiofemoral joint was driven by marker trajectories, and the other 10 DOFs were determined by an FDK algorithm. For each simulation step, the FDK algorithm searched iteratively the secondary movements ($\alpha^{FDK}$) until quasi-static equilibrium was achieved [13]. In our model, the threshold of $F^{FDK}$ was set to 1 N.

The simulation process of our proposed musculoskeletal model is summarized as PI (parameter identification), MT (marker tracking), and ID (inverse dynamics). Firstly, PI was applied to scale the model as described in the previous section. The scaled model is shown in Figure 3D. Secondly, MT was utilized to track the marker trajectories and calculate the knee kinematics. Finally, the GRFs and MT results were input into ID to compute TFCF_Total_, TFCF_Medial_, and TFCF_Lateral_ (the resultant force in three anatomical directions).

### 2.4. Statistical Analysis

All TFCFs were expressed as mean ± standard deviation (SD) and in the unit of BW (body weight), and were resampled on a 0% to 100% normalized time. To assess the prediction accuracy, the comparison of the entire TFCF curves between the predictions based on our proposed musculoskeletal model incorporated with a subject-specific knee joint and in vivo measurements based on the instrumented knee prostheses during the five daily activities was performed in this study. The predicted TFCF curves were obtained from the 18 healthy participants recruited in this study, and the in vivo measured TFCF curves were obtained from a male participant who had undergone TKA (weight: 77 kg, height: 1.74 m, and BMI: 25.43 kg/m^2^) [36]. For each of the five daily activities, the prediction accuracy was quantified in terms of the Root Mean Square Error (RMSE), Coefficient of Determination (R^2^), and Sprague and Geers metrics of combined error (C_E_). Among them, the C_E_ has been validated and is often used to compare and analyze predicted and measured curves in biomechanical research [37].

After the proposed musculoskeletal model evaluation, the TFCF_Total_, TFCF_Medial_, and TFCF_Lateral_ during the five daily activities were predicted for all of the 18 healthy participants. The peak TFCF values were not normally distributed after the normality was checked by the Shapiro–Wilk test. The comparison between the peak TFCF values was performed using the non-parametric Wilcoxon signed-rank test, and a *p*-value less than 0.05 was considered statistically significant. Statistical analyses were performed using SPSS software (Version 19, Chicago, IL, USA).

## 3. Results

**Figure 4:**
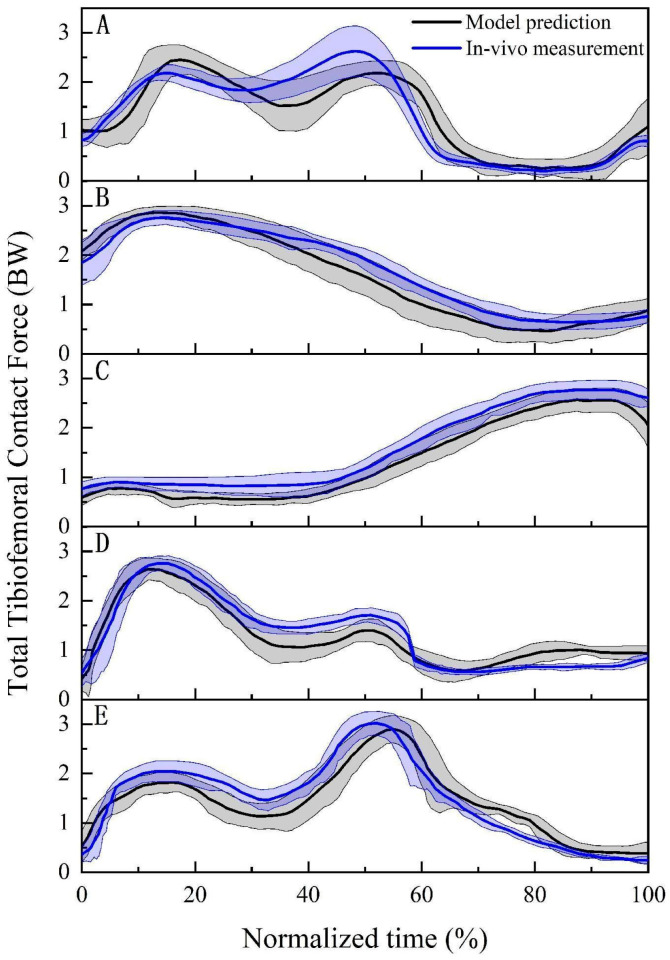
Comparisons of the entire tibiofemoral contact force (TFCF) curves (mean (solid lines) and standard deviation (shaded regions)) between the predictions based on our musculoskeletal model and in vivo measurements based on the instrumented prostheses during (**A**) normal walking, (**B**) sit-to-stand, (**C**) stand-to-sit, (**D**) stair ascent, and (**E**) stair descent.

The comparisons of entire TFCF curves between the prediction based on our proposed musculoskeletal model incorporated with a subject-specific knee joint and in vivo measurement based on the instrumented prostheses during the five daily activities are presented in Figure 4. The prediction accuracies of our proposed musculoskeletal model were as follows: normal walking: RMSE 0.311, R^2^ 0.928, and C_E_ 0.080; sit-to-stand: RMSE 0.216, R^2^ 0.977, and C_E_ 0.048; stand-to-sit: RMSE 0.241, R^2^ 0.992, and C_E_ 0.141; stair ascent: RMSE 0.261, R^2^ 0.936, and C_E_ 0.082; stair descent: RMSE 0.290, R^2^ 0.946, and C_E_ 0.089. In general, our proposed musculoskeletal model incorporated with a subject-specific knee joint was able to predict the TFCT with a reasonable accuracy for all the five daily activities (RMSE: 0.216~0.311 BW, R^2^: 0.928~0.992, and C_E_: 0.048~0.141).

**Figure 5:**
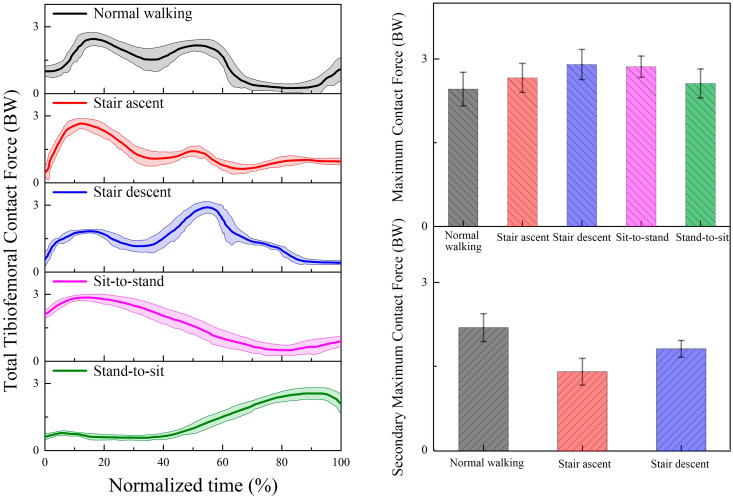
The predicted tibiofemoral contact force (mean (solid lines) and standard deviation (shaded regions)) on the total compartment during normal walking, stair ascent, stair descent, sit-to-stand, and stand-to-sit.

**Figure 6:**
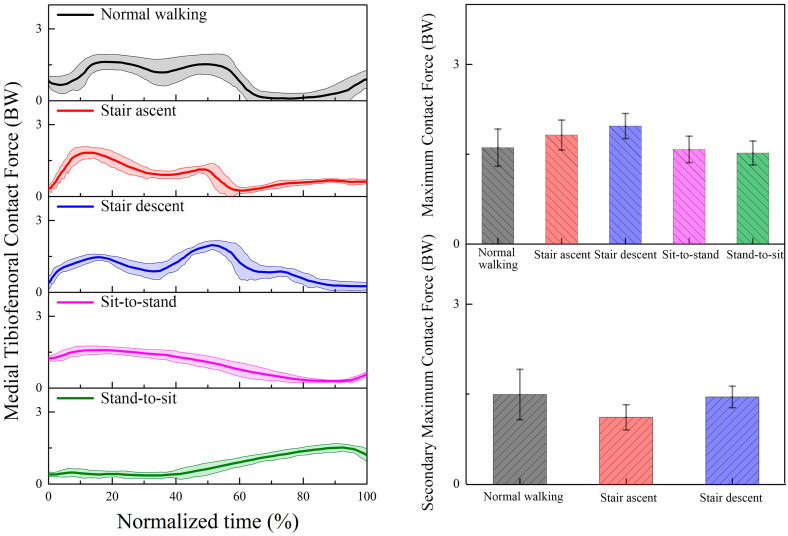
The predicted tibiofemoral contact force (mean (solid lines) and standard deviation (shaded regions)) on the medial compartment during normal walking, stair ascent, stair descent, sit-to-stand, and stand-to-sit.

**Figure 7:**
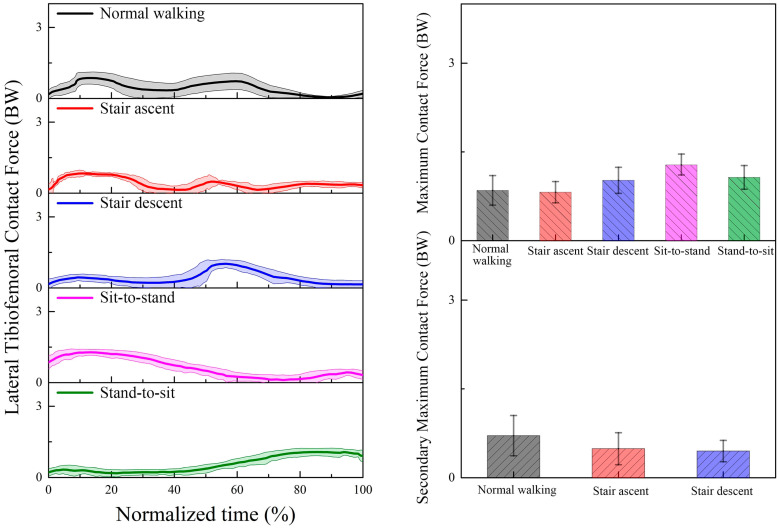
The predicted tibiofemoral contact force (mean (solid lines) and standard deviation (shaded regions)) on the lateral compartment during normal walking, stair ascent, stair descent, sit-to-stand, and stand-to-sit.

The predicted TFCF_Total_, TFCF_Medial_, and TFCF_Lateral_ during the five daily activities based on our proposed musculoskeletal model incorporated with a subject-specific knee joint are presented in Figure 5, Figure 6 and Figure 7. For normal walking, double peaks are observed for both TFCF_Total_, TFCF_Medial_, and TFCF_Lateral_ curves. The first peaks were 2.46 ± 0.30 BW, 1.61 ± 0.31 BW, and 0.85 ± 0.25 BW in the early stance, and the second peaks were 2.21 ± 0.25 BW, 1.50 ± 0.42 BW, and 0.71 ± 0.34 BW in the terminal stance for TFCF_Total_, TFCF_Medial_, and TFCF_Lateral_, respectively. For all TFCF curves, the first peaks are significantly larger than the second peaks (*p* < 0.05). The medial compartment accounted for nearly 65% of the TFCF_Total_ during the whole cycle. For the stair ascent or stair descent activity, double peaks are found for all TFCF_Total_, TFCF_Medial_, and TFCF_Lateral_ curves. Comparing the two peaks of TFCF_Total_, TFCF_Medial_, and TFCF_Lateral_, stair ascent exhibits larger first peaks in the early stance (first peak: 2.66 ± 0.26 BW, 1.82 ± 0.25 BW, and 0.84 ± 0.18 BW; second peak: 1.42 ± 0.24 BW, 1.12 ± 0.21 BW, and 0.49 ± 0.27 BW), while stair descent exhibits larger second peaks in the terminal stance (first peak: 1.83 ± 0.15 BW, 1.46 ± 0.18 BW, and 0.45 ± 0.18 BW; second peak: 2.90 ± 0.27 BW, 1.97 ± 0.21 BW, and 1.02 ± 0.22 BW) (*p* < 0.01). Comparing TFCF_Medial_ and TFCF_Lateral_, TFCF_Medial_ accounts for almost 68% of the TFCF_Total_ for stair ascent and 70% for stair descent. For the sit-to-stand or stand-to-sit activity, a single peak is shown for all TFCF_Total_, TFCF_Medial_, and TFCF_Lateral_ curves. The peaks occur in the early phase for sit-to-stand and the terminal phase for stand-to-sit. For TFCF_Total_, the peak is 2.86 ± 0.19 BW for sit-to-stand and 2.56 ± 0.26 BW for stand-to-sit. The medial compartment still bore more loads (55% and 59%) than the lateral compartment during the whole movement. For the peaks TFCF_Medial_ and TFCF_Lateral_, 1.58 ± 0.22 BW and 1.28 ± 0.17 BW are observed for sit-to-stand, and 1.52 ± 0.20 BW and 1.07 ± 0.20 BW are observed for stand-to-sit. When comparing the five daily activities, the peak TFCF_Total_ of stair descent or sit-to-stand is the largest, followed by stair ascent or stand-to-sit, and finally normal walking (*p* < 0.05). For the medial compartment, stair descent has the largest peak TFCF_Medial_, followed by stair ascent. There are no significant differences between the peak TFCF_Medial_ of normal walking, sit-to-stand, and stand-to-sit (*p* > 0.05). For the lateral compartment, the peak TFCF_Lateral_ of sit-to-stand is the largest, followed by stand-to-sit or stair descent, and finally normal walking or stair ascent (*p* < 0.05).

## 4. Discussion

Accurate quantification of internal knee contact force during daily activities is important for comprehending KOA initiation and progression, and improving KOA treatments. Although various musculoskeletal models have been previously developed to predict the TFCF, previous studies have been limited by the lower degree of personalization of the models and the fewer daily activities predicted simultaneously by the models. The aim of this study was to develop a musculoskeletal model incorporated with a subject-specific knee joint to predict the TFCF_Total_, TFCF_Medial_, and TFCF_Lateral_ during the five daily activities. The results showed that our proposed musculoskeletal model could predict the TFCF during the five daily activities with a reasonable accuracy.

The prediction accuracy of our proposed musculoskeletal model was assessed by comparing the entire TFCF curves between the predictions and in vivo measurements [36]. The comparative results showed that the predicted TFCF curve patterns for the five activities were similar to those measured in vivo. In terms of prediction accuracy, our predictions were in good agreement with the in vivo measurement for all of the five activities (normal walking: RMSE 0.311, R^2^ 0.928, and C_E_ 0.080; sit-to-stand: RMSE 0.216, R^2^ 0.977, and C_E_ 0.048; stand-to-sit: RMSE 0.241, R^2^ 0.992, and C_E_ 0.141; stair ascent: RMSE 0.261, R^2^ 0.936, and C_E_ 0.082; stair descent: RMSE 0.290, R^2^ 0.946, and C_E_ 0.089). There were two main potential factors that could have negatively affected the prediction accuracy. Firstly, the predicted TFCF curves were obtained from the healthy individuals, while the in vivo measured TFCF curves were obtained from a participant after TKA. Secondly, the experimental equipment was different between our prediction and in vivo measurement. In our study, a 0.44 m chair was used for sit-to-stand and stand-to-sit, and a three-step staircase (0.29 m tread and 0.17 m riser) was used for stair ascent and descent, which is inconsistent with the equipment used for in vivo measurement. According to the studies of Janssen et al. [38] and Trinler et al. [39], the chair height and the stair dimension had significant effects on the TFCF during sit-to-stand/stand-to-sit and stair ascent/descent, respectively. In addition, the prediction accuracy of our musculoskeletal model was similar to the previous studies (Lerner et al. [7]: RMSE 0.34 BW; Moissenet et al. [9]: RMSE 0.39 BW; Smith et al. [11]: RMSE 0.33 BW; Kia et al. [12]: RMSE 0.32~0.68 BW; Marra et al. [14]: RMSE 0.26 BW; Thelen et al. [40]: RMSE 0.51 BW; Chen et al. [41]: RMSE 0.27~0.45 BW). Overall, our proposed musculoskeletal model incorporated with a subject-specific knee joint could predict the TFCF_Total_, TFCF_Medial_, and TFCF_Lateral_ during the five activities with reasonable accuracy, which is consistent with our hypothesis.

Based on our proposed and validated musculoskeletal model incorporated with a subject-specific knee joint, the TFCF_Total_, TFCF_Medial_, and TFCF_Lateral_ during five activities, including normal walking, stair ascent, stair descent, sit-to-stand, and stand-to-sit, were predicted in this study. The results showed that the TFCF_Total_, TFCF_Medial_, and TFCF_Lateral_ curves had similar curve patterns, i.e., two peaks for normal walking, stair ascent, and stair descent, and one peak for sit-to-stand and stand-to-sit, between our predictions and the predictions of previous studies [15,16,17]. For stair ascent, the two peaks of TFCF_Total_ predicted by our model were smaller than those by the models of Konrath et al. [17] (first peak: 3.48 ± 0.21 BW; 2.55 ± 0.77 BW), and Bennett et al. [42] (first peak: 6.16 ± 0.69 BW; second peak: 2.90 ± 0.29 BW). For stair descent, the two peaks of TFCF_Total_ predicted by our model were smaller than those by the models of Konrath et al. [17] (first peak: 3.53 ± 0.54 BW; second peak: 4.39 ± 0.60 BW). Compared with our subject-specific musculoskeletal model, the model of Lerner et al. [7] incorporated with subject-specific tibiofemoral alignment and medial–lateral contact locations was applied in the study of Bennett et al. [42], and the generic musculoskeletal model was utilized in the study of Konrath et al. [17]. The degree of personalization of the models in these studies was lower than our musculoskeletal model incorporated with a subject-specific knee joint, which may increase the predicted TFCF in these studies. Consistent with the previous studies [15,16,17], this study also found that stair ascent produced larger first peaks in the early stance, while stair descent produced larger second peaks in the terminal stance. For sit-to-stand and stand-to-sit, the peaks of TFCF_Total_ predicted by our model were smaller than those by the models of Navacchia et al. [43] (stand-to-sit: 3.30 ± 0.20 BW; sit-to-stand: 3.12 ± 0.70 BW) and Smith et al. [44] (sit-to-stand: 3.17 ± 0.76 BW). Navacchia et al. [43] established a subject-specific musculoskeletal model with linear scaling bones, a one-DOF knee joint, and no ligaments. Smith et al. [44] utilized the FreeBody model proposed by Cleather and Bull [45]. The greater peak of TFCF of these studies may be due to the lower personalization degree of their musculoskeletal model. When comparing the five activities, our finding showed that the peak TFCF_Total_ of stair descent or sit-to-stand was the largest, followed by stair ascent or stand-to-sit, and finally normal walking, which was consistent with previous studies [46]. However, the stair descent had the largest peak TFCF_Medial_, followed by stair ascent, and finally normal walking, sit-to-stand, or stand-to-sit. The order of peak TFCF_Lateral_ from high to low was sit-to-stand, stand-to-sit or stair descent, and normal walking or stair ascent. The reasons may be twofold. Firstly, our results showed that the medial compartment accounted for almost 65% of the TFCF_Total_ during normal walking, 68% during the stair ascent, 70% during stair descent, 55% during sit-to-stand, and 59% during stand-to-sit. The greater ratio could increase the load on the medial compartment and decrease the load on the lateral compartment, which could then change the order of the peak TFCF_Medial_ and the peak TFCF_Lateral_. Secondly, the peaks of normal walking, stair ascent, and stair descent were observed at the transition time between double-leg support and single-leg support, while the body was supported by both legs throughout the sit-to-stand and stand-to-sit activities. Thirdly, the greater knee flexion angles may have increased the TFCF_Total_ and TFCF_Medial_ [47]. According to our previous study [48], the knee flexion ROM (range of motion) of sit-to-stand or stand-to-sit was the largest, followed by stair ascent and stair descent, followed by normal walking.

Several limitations of this study should be acknowledged. Firstly, the TFCF values were calculated by using the linear force–penetration volume law, with the material properties of cartilages and the meniscus assumed to follow linear elasticity. The subject-specific model with more realistic material properties of cartilages and the meniscus needs to be studied in more detail. Secondly, the relative movement between meniscus and tibia was ignored in our study. Vedi et al. [49] studied the in vivo movement of the meniscus as it moved from knee full extension to 90 deg knee flexion while weight bearing by using dynamic MRI. The findings showed that the anterior and posterior horns of the medial meniscus moved 7.1 mm and 3.9 mm, with 3.6 mm of radial displacement, respectively, while the anterior and posterior horns of the lateral meniscus moved 9.5 mm and 5.6 mm, with 3.7 mm of radial displacement, respectively. The effect of the relative movement between meniscus and tibia on the prediction of TFCF is unclear and needs further study. Thirdly, the attachment points and mechanical properties of the knee ligaments, as referenced in previous studies, were identified rather than using personalized data, which might have influenced the prediction accuracy of TFCF. Finally, in order to simplify the simulation process, the patellar tendon was defined as rigid material. The stiffness of the patellar tendon was about 14,700 N/strain, which was stiffer than other knee ligaments [11]. The effect of the rigid patellar tendon on the prediction of TFCF will be discussed in the future.

## 5. Conclusions

In this study, we proposed a musculoskeletal model incorporated with a subject-specific knee joint to predict the TFCF during the five daily activities. The prediction accuracy of our proposed model was demonstrated by the good agreements of entire TFCF curves between the predictions and the in vivo measurements for all of the five daily activities. Based on the model, the TFCF_Total_, TFCF_Medial_, and TFCF_Lateral_ during the five daily activities were predicted. For the total compartment, the peak of stair descent or sit-to-stand was the largest, followed by stair ascent or stand-to-sit, and finally normal walking. For the medial compartment, the stair descent had the largest peak, followed by stair ascent. There were no significant differences between the peak TFCF_Medial_ of normal walking, sit-to-stand, and stand-to-sit. For the lateral compartment, the peak of sit-to-stand was the largest, followed by stand-to-sit or stair descent, and finally normal walking or stair ascent. This study may have significant clinical implications for understanding the biomechanics of natural knee joint and providing theoretical guidance for the treatments of KOA, such as knee brace, gait modification, and TKA surgery.

## Figures and Tables

**Table 1:** Parameters (reference strain ($\varepsilon_{r}$) and stiffness (*k*)) of the ligament bundles [14,29,30].

Ligament	Bundle	k (N/Strain)	$\varepsilon_{r}$
ACL (anterior cruciate ligament)	aACL (anterior bundle)	5000	0.06
	pACL (posterior bundle)	5000	0.10
PCL (posterior cruciate ligament)	aPCL (anterior bundle)	9000	−0.24
	pPCL (posterior bundle)	9000	−0.03
LCL (lateral collateral ligament)	aLCL (anterior bundle)	2000	0.03
	mLCL (middle bundle)	2000	−0.05
	pLCL (posterior bundle)	2000	0.08
MCL (medial collateral ligament)	aMCL (anterior bundle in superior layer)	2500	0.04
	mMCL (middle bundle in superior layer)	3000	0.04
	pMCL (posterior bundle in superior layer)	2500	0.04
	aDMCL (anterior bundle in deep layer)	1000	−0.18
	pDMCL (posterior bundle in deep layer)	1000	−0.04
MPFL (medial patellofemoral ligament)	sMPFL (superior bundle)	2000	0.08
	mMPFL (middle bundle)	2000	0.08
	iMPFL (inferior bundle)	2000	0.08
LPFL (lateral patellofemoral ligament)	sLPFL (superior bundle)	2000	0.06
	mLPFL (middle bundle)	2000	0.06
	iLPFL (inferior bundle)	2000	0.06
PMC (posteromedial complex)		2000	−0.04
POL (posterior oblique ligament)		1500	−0.18
ALL (anterolateral ligament)		2000	0.05
PFL (popliteofibular ligament)		2000	0.05

**Table 2:** Referenced *v*, *E*, and *h* of the contact objects in the natural knee model [34,35].

Contact Object	Poisson’s Ratio (*v*)	Elasticity Modulus (*E*, MPa)	Average Elastic Layer Thickness (*h*, mm)
Femoral cartilage	0.46	5	2.2
Medial tibial cartilage	0.46	5	2.1
Lateral tibial cartilage	0.46	5	2.6
Medial meniscus	0.49	59	3.5
Lateral meniscus	0.49	59	3.2
Patellar cartilage	0.46	5	3.3

## Data Availability

All data used in this study are available from the corresponding author on reasonable request.
